# Supplementary material for: Effects of Voluntary Physical Exercise on the Neurovascular Unit in a Mouse Model of Alzheimer’s Disease
Source: Int J Mol Sci. 2023 Jul 6;24(13):11134. doi: 10.3390/ijms241311134 (PMC10342693; doi:10.3390/ijms241311134)
Supplement: Supplementary file 1 [file ijms-24-11134-s001.zip › supplementary figure.pdf]

Supplementary Materials

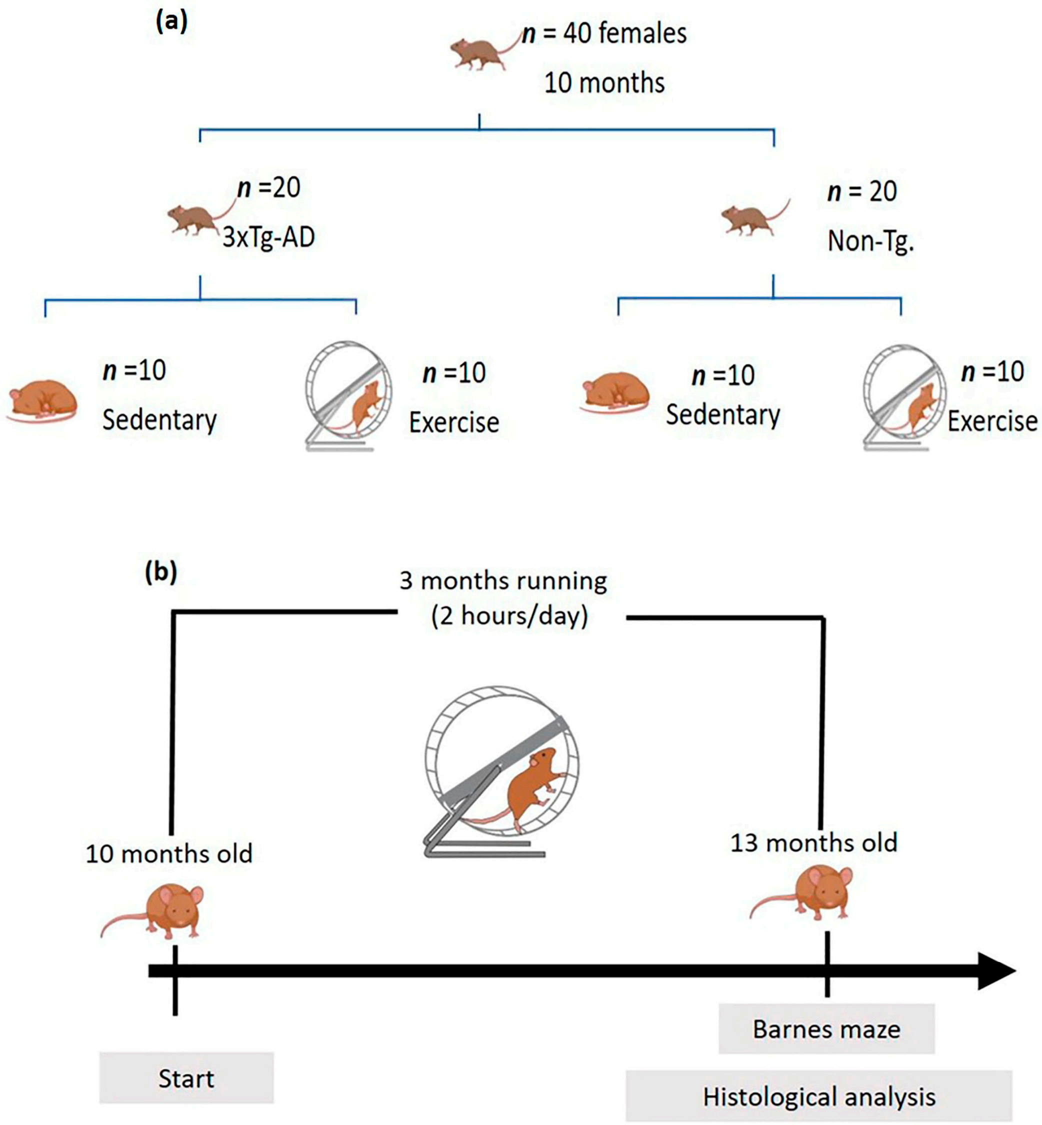

Figure S1. Illustration of experimental design. (**a**) Number of animals used per group and condition. (**b**) Voluntary physical exercise regimen and behavioral/histological analysis.
